# Bilateral inguinoscrotal swelling: An uncommon presentation of omental cystic lymphangioma

**DOI:** 10.12669/pjms.40.5.8255

**Published:** 2024

**Authors:** Sana Viqar, Chandni, Muhammad Amjad Chaudhary

**Affiliations:** 1 Sana Viqar, Department of Pediatric Surgery, The Childrens Hospital, Pakistan Institute of Medical Sciences, Islamabad, Pakistan; 2 Chandni, Department of Pediatric Surgery, The Childrens Hospital, Pakistan Institute of Medical Sciences, Islamabad, Pakistan; 3 Muhammad Amjad Chaudhary, MS, FRCS(Edin), FRCS (Glasgow), Professor and Head of department, Department of Pediatric Surgery, The Childrens Hospital, Pakistan Institute of Medical Sciences, Islamabad, Pakistan

**Keywords:** Omental cystic lymphangioma, Inguinoscrotal swelling, Communicating hydrocele

## Abstract

Abdominal cystic lymphangioma is a rare benign tumour in children. It is often difficult to diagnosis pre-operatively due to a varied spectrum of symptoms. We report a case of a male infant who presented with gross bilateral inguinoscrotal swelling. Provisional diagnosis of congenital communicating hydrocele was made and investigation revealed a large abdominal cyst. Patient underwent explorative laparotomy and the cyst arising from greater omentum, extending into bilateral scrotum, was excised and bilateral herniotomy done. Mass was confirmed to be lymphangioma on biopsy. This case is unique as an abdominal lymphangioma presented solely as inguinoscrotal swelling, with no abdominal symptom. To our knowledge, this is the first case of omental cystic lymphangioma involving both inguinoscrotal regions. Our case suggests that abdominal cystic lymphangioma should be a part of the differential diagnosis in any child with gross inguinoscrotal swelling in whom initial impression is of communicating hydrocele.

## INTRODUCTION

Cystic Lymphangioma is a rare benign hamartoma arising from the maldevelopment of the lymphatic system. While neck and axilla are the most common locations of lymphangioma in children, abdominal origin is rare^1^ and this includes namely mesenteric and omental cystic lymphangiomas.

Diagnosis of abdominal lymphangioma is often challenging due to a varied spectrum of presenting symptoms. Complete surgical excision remains the gold standard treatment and final diagnosis is confirmed on histopathology.^2^ We present a case of an infant with an abdominal lymphangioma with an uncommon presentation of bilateral inguinoscrotal swelling since birth.

## CASE REPORT

An 11 months old male infant presented to The Children Hospital, PIMS, Islamabad in March 2023 with bilateral inguinoscrotal swelling since birth. Parents noticed a gradual increase in size of the swelling over the last four months. The swelling was not associated with inguinoscrotal pain, urinary symptoms, abdominal distention or symptoms of obstruction. There was no significant past medical or surgical history. Child was breast fed, weaned at six months of life, vaccinated and developmental milestones were achieved according to age. His vitals were within normal range.

Local examination revealed gross bilateral inguinoscrotal swelling which was soft, non-tender having a smooth surface with normal overlying skin. The swelling was compressible, non-reducible, positively trans illuminant and upper margins were not reachable. Testes on both sides could not be separately palpated. Abdomen was soft, mildly distended with no palpable mass. His initial workup showed Hemoglobin of 7.6g/dl. Serum electrolytes, renal and liver function test were within normal range.

**Fig.1:**
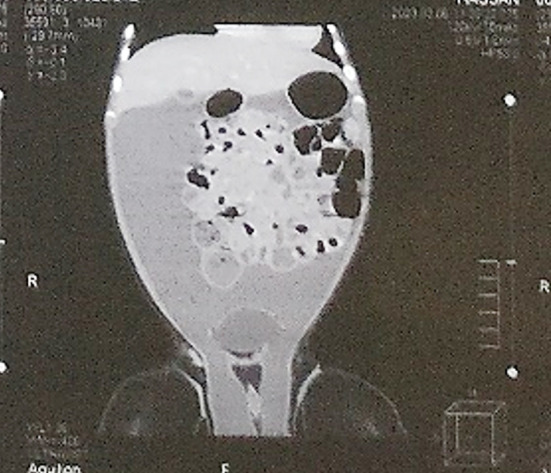
CT film showing extent of intraabdominal cystic lymphangioma into both scrotal regions.

Ultrasound of bilateral inguinoscrotal region showed evidence of free fluid in bilateral scrotal cavities, with fluid communicating with peritoneal cavity, suggestive of bilateral gross congenital hydrocele. Both testes of normal size and echotexture were seen in scrotum. CT Abdomen and pelvis with contrast showed a huge hypodense cystic 15 x 11 cm with hyperdense multiple internal septation, having no solid component. It was extending through bilateral inguinal canal into scrotum, with extension along right paracolic gutter into subhepatic region of the right side, shown in Fig.1. All these features were in favor of abdominal lymphatic malformation.

**Fig.2:**
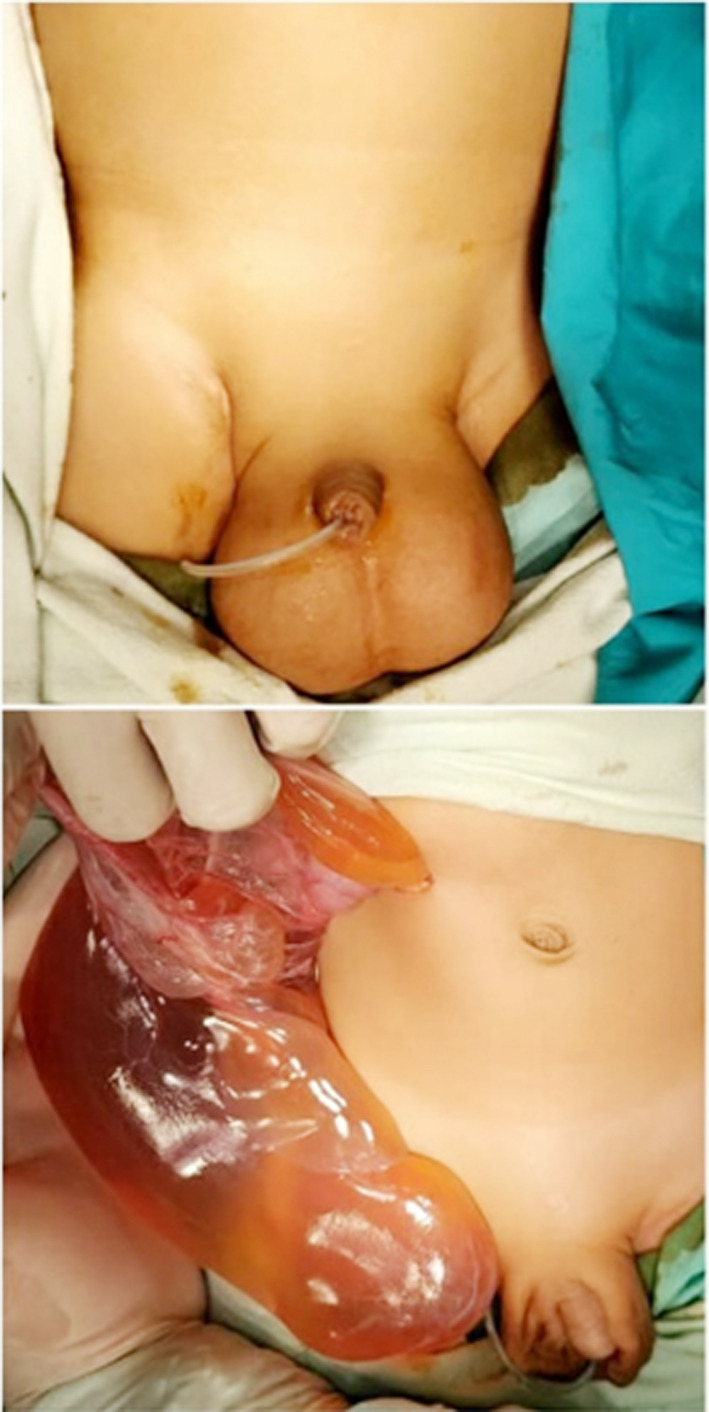
Gross bilateral inguinoscrotal swelling and intraoperative finding of the cyst.

Patient was transfused red cell concentrates and prepared for explorative laparotomy. Right upper transverse skin incision was made. On opening the peritoneum, a large cyst was noted. It was seen arising from greater omentum approximately 12x15cm with multilocation containing straw colored fluid as shown in Fig.2. The cyst was present from subhepatic space up to the scrotum. It was not adherent to bowel or any other structure. Cyst was gently mobilized from the scrotal space and completely excised. Bilateral hernial orifices were closed at the level of deep ring using purse string. Post-operative recovery was uneventful and patient was discharged on 4^th^ post-operative day. Histopathology report showed a thin cyst lined by cuboidal to low columnar epithelium, connective tissue stroma containing various size of lymphoids, smooth muscles and inflammatory cells. This confirmed the diagnosis of omental cystic lymphangioma.

## DISCUSSION

Lymphangioma results from the maldevelopment of primordial lymphatic sacs, often having no connection with the normal lymphatic system. Lymphangiomas comprise of 6% of all benign tumors in the pediatric population and among them abdominal origin accounts for 3-9%.^2^

Abdominal omental cysts have been reported in literature with various presentation, such as abdominal distension, pain and ascites. One study from Pakistan reported a case of acute abdomen in a child with hemoperitoneum; explorative laparotomy revealed a large omental cyst with spontaneous massive hemorrhage.^3^ In one study conducted over 26 years, 13 cases of pediatric abdominal lymphangioma were reported out of which abdominal pain and mass were the most common symptoms.^4^ In another 10 years study, 45 cases of lymphangioma were encountered, out of which only seven arose from rare sites like bowel mesentery, retroperitoneum, inguinal and gluteal region. They reported one case of cystic lymphangioma extending into inguinoscrotal region, similar to our case but arising from the retroperitoneum.^1^ One other study reported an extension of retroperitoneal lymphangioma into the inguinoscrotal region. Two separate abdominal and scrotal incisions were made to perform a subtotal excision of mass.^5^ Rarely, inguinoscrotal swelling can be cystic lymphangioma of scrotal origin, mimicking as hydrocele.^6^,^7^

One study most similar to the case we have reported was a case of an abdominal cystic lymphangioma presenting as left sided inguinoscrotal swelling and abdominal distension. In this case retroperitoneal approach was initially taken but upon finding no cyst in retroperitoneal space, peritoneum was opened to find the cyst arising from greater omentum.^8^ One study reported a misdiagnosed case of hydrocele in which inguinal approach of hydrocele repair was taken. Diagnosis of abdominal lymphangioma was made manifest postoperatively due to hemorrhage within the cyst.^9^ This highlights the importance of keeping abdominal cystic lymphangioma as a differential in gross communicating hydrocele.

Initial choice of investigation for abdominal lymphangioma is ultrasound which suggests multiloculated cystic appearance. CT scan can be done to assess the origin, size and relationship of cyst with other structures. Recent studies have demonstrated successful resection of abdominal lymphatic malformation using laparoscopy.^10^,^11^ While complete resection of large masses may be difficult, having a risk of recurrence, promising results shown by laparoscopic approach makes it an ideal choice of approach. In our case as CT showed a very large cystic mass occupying the whole right hemiabdomen, decision to do laparotomy was made based on anticipated technical challenges.

The case we present is unique as the patient with an abdominal lymphangioma presented solely with inguinoscrotal swelling, and no abdominal symptom. This is perhaps the first case of omental cystic lymphangioma involving both inguinoscrotal regions as we could not find any other study during literature search.

## CONCLUSION

A rare condition should be kept in the differential diagnosis of a common presentation. Omental cystic lymphangioma may often be mistaken as congenital hydrocele. This case report suggests that in any child with gross swelling of inguinoscrotal region, unilateral or bilateral, an index of suspicion of an abdominal cystic mass must be present so that relevant investigations are advised.

### Authors’ Contribution:

**SV:** Data acquisition, Manuscript writing, Literature review. Is responsible and accountable for the accuracy and integrity of the work.

**Chandni:** Literature review, Manuscript editing.

**MAC:** Conception and design of study, Supervision, Review and Final approval.
